# The Combination of T Stage and the Number of Pathologic Lymph Nodes Provides Better Prognostic Discrimination in Early-Stage Cervical Cancer With Lymph Node Involvement

**DOI:** 10.3389/fonc.2021.764065

**Published:** 2021-11-05

**Authors:** Yongrui Bai, Ling Rong, Bin Hu, Xiumei Ma, Jiahui Wang, Haiyan Chen

**Affiliations:** Department of Radiation Oncology, Renji Hospital, Shanghai Jiao Tong University, School of Medicine, Shanghai, China

**Keywords:** cervical cancer, lymph node, sequential chemoradiotherapy, T stage, N stage, radical hysterectomy

## Abstract

**Objective:**

Stage I and II cervical cancer with pelvic and/or para-aortic lymph node (LN) metastases are upstaged to stage IIIC under the new FIGO 2018 staging system, and radical chemoradiotherapy was recommended. But heterogeneity in outcome existed in this group of patients. We conducted this retrospective analysis to evaluate the heterogeneity of these patients and tried to provide a more detailed classification to reflect the prognosis and guide the treatment. We also evaluated the efficacy and toxicity of surgery followed by sequential chemoradiotherapy in this cohort.

**Methods:**

Early-stage cervical cancer with LN involvement that had radical hysterectomy followed by sequential chemoradiotherapy were retrospectively analyzed. Survival analyses were conducted to identify the prognostic factors.

**Results:**

A total of 242 patients were included in the study; 64 (26.4%) patients had treatment failure, and 51 (21.1%) died. Pathology, T stage, the number of pathologic LN (pLN), and neoadjuvant chemotherapy or not were independent prognostic factors for disease-free survival and overall survival (OS). Patients with T1N < 3 pLN had significantly better survival than T2N < 3 pLN/T1-2 N≥ 3 pLN, with failure rates of 11.6% and 35.8% in each group; and 5 year OS was 92% and 62%, respectively (P = 0.000). About 1.5% of the patients discontinued radiotherapy, and 14.1% had G3-4 hematological toxic effects during radiotherapy; 1.7% developed G2-3 lower limb edema, and 2.9% developed severe urinary toxicity.

**Conclusion:**

Nodal involvement alone is inadequate as the sole pathologic factor to predict survival in early-stage cervical cancer. The combination of tumor and node subcategory provides better prognostic discrimination.

## Introduction

Cervical cancer continues to be a major public health problem affecting middle-aged women. It ranked in the top three cancers affecting women younger than 45 years. China and India together contributed more than a third of the global cervical burden, with 106,000 cases in China and 97,000 cases in India, and 48,000 deaths in China and 60,000 deaths in India (1). One of the most important prognostic indicators in cervical cancer is the lymph node (LN) status. In order to highlight the importance of nodal involvement, patients who are previously International Federation of Gynecology and Obstetrics (FIGO) stage I and II, with the presence of pelvic and/or para-aortic lymph node metastases, are upstaged to stage IIIC under the new FIGO 2018 staging system (2, 3). And treatment recommendation is provided tailored to the new classification. Current European guidelines suggest concurrent chemoradiotherapy (CCRT) in early-stage disease with pelvic positive lymph nodes (pLN) on radiological staging; in cases where positive nodes (macrometastasis or micrometastasis) are detected intraoperatively, surgery should be aborted and switched to definitive chemoradiotherapy (4). In the latest version of the NCCN guideline, for patients with positive pelvic and/or para-aortic lymph nodes, the preferred treatment consists of pelvic EBRT with concurrent platinum-containing chemotherapy and brachytherapy (5). Some authors, however, have revisited their series in light of the new FIGO staging system, engendering some questions concerning its reliability. It is found that women with stage IIIC1 had superior cervical cancer-specific survival compared to those with stage IIIA-B disease, and varied significantly depending on various factors, such as T-stage, LN burden, etc. (3, 6–8). Nodal involvement alone seems inadequate as the sole pathologic factor to predict survival and relapse rates. Meanwhile, for early-stage cervical cancer with LN involvement, treatment is variable in clinics, with radical surgery remaining the prevailing management strategy (9), and concurrent chemoradiotherapy following surgery is often scheduled. In our department, sequential chemoradiotherapy (SCRT) was given to these patients following radical hysterectomy. We conducted this retrospective study, focused on early-stage cervical cancer with LN involvement, to evaluate the heterogeneity in this group of patients and tried to provide a more detailed classification to reflect the prognosis. We also evaluated the efficacy and toxicity of surgery followed by SCRT in this cohort.

## Materials and Methods

### Inclusion and Exclusion

The present study was approved by the Institutional Review Board of Renji Hospital. We reviewed the medical records of all early-stage uterine cervical cancer patients who received adjuvant radiotherapy (RT) at our institution between 2010 and 2019. Inclusion criteria were as follows: (1) diagnosis of invasive cervical cancer, T_1-2_ at presentation; (2) radical hysterectomy plus pelvic lymphadenectomy (with or without para-aortic lymphadenectomy); (3) with pathologic confirmed pelvic (with LN identified on preoperative image or not); (6) consolidation chemotherapy; (7) neoadjuvant chemotherapy before surgery or not. Patients whose T1-2 tumor was considered to be borderline resectable due to being a large tumor would be given neoadjuvant chemotherapy; for patients whose tumor became smaller (at least partial response after neoadjuvant chemotherapy) and converted to be resectable, radical surgery was given. Exclusion criteria were as follows: (1) with gross residual disease after surgery; (2) pT3-4 disease; (3) synchronous malignancies (within 5 years); (4) patients with para-aortic LN metastasis.

### Treatment and Follow-Up

Adjuvant RT with three-dimensional conformal RT or intensity modulated radiotherapy (IMRT) was started with a median of 39 (28-83) days after surgery. Before 2017, for patients without para-aortic LN metastasis, the clinical target volume (CTV) included the common iliac vessel, external and internal iliac vessels, presacral area, parametrium, and upper vagina, in accordance with the CTV guidelines by the Radiation Therapy Oncology Group for whole pelvis RT. For all patients with para-aortic LN metastasis, the para-aortic area was irradiated. Since 2017, for patients with positive common iliac LN or more than two pLNs in the pelvis, extended field irradiation was also given. The median radiation dose was 50.4 Gy, ranging from 45.0 to 50.4 Gy in 23–28 fractions (1.8–2 Gy per fraction). For patients with a positive parametrium margin, an addition of 5–15 Gy was given, depending on normal tissue. Intracavitary brachytherapy was indicated for patients with a close (≤ 5 mm) or positive vaginal resection margin, with a total dose of 10–24 Gy in two to four fractions. Chemotherapy (sequential or concurrent with radiotherapy) was given depending on the doctor in-charge.

Follow-up evaluations were scheduled every 3 months for the first 2 years after radiotherapy, every 6 months between 2 and 5 years after radiotherapy, and annually thereafter. Survival data were abstracted from a dedicated database updated on a regular basis. Recurrence was classified as locoregional if detected in the pelvic region, as para-aortic if detected in the para-aortic area, and as distant if detected outside the pelvic and para-aortic region. Toxicity was graded referring to the National Cancer Institute Common Terminology Criteria for Adverse Events (CTCAE) 5.0. Acute treatment toxicities were regularly recorded during treatment, and late toxicities were collected and graded retrospectively. Rigorous efforts, including telephone interviews, were done to improve the quality of follow-up data.

### Statistical Analyses

Data were summarized using standard descriptive statistics. Analyses were performed using SPSS 22.0. Continuous variables were compared by Student’s t-tests; categorical variables were analyzed using Pearson’s chi-square tests or Fisher’s exact tests. Survival analysis was conducted using the Kaplan–Meier method with log-rank tests. Univariable Cox regression analysis was used to identify risk factors of disease-free survival (DFS) and overall survival (OS). All p values were two-sided; values of p < 0.05 were considered statistically significant.

## Results

### Patient Characteristic

Overall, 310 cervical cancer patients were diagnosed with pLN; after excluding patients with R2 resection, with para-aortic LN metastasis, lost-of follow-up, or without adjuvant chemotherapy, 242 patients were included in this analysis. **Table 1** shows the disease- and treatment-related characteristics. A total of 124 patients had pelvic MRI and abdomen CT before treatment, 24 patients had PET-CT and among them 12 also had pelvic MRI, 91 patients had pelvic and abdomen CT, while the remaining 3 patients only had endovaginal ultrasound. A total of 60 patients were diagnosed with metastatic LN before treatment.

**Table 1:** Patient and treatment characteristics.

Characteristics	NO	Characteristics	NO
Age (years)	50 ± 10.9	LVSI	
Tumor size at presentation	4.4 ± 3.7	Yes	166 (68.6%)
Surgical approach		No	76 (31.4%)
Open	36 (14.9%)	Margin status	
Laparoscopic	206 (85.1%)	≥5mm	206 (85.1%)
Pathology		<5mm	36 (14.9%)
SCC	209 (86.4%)	Parametrial invasion	
Non-SCC	33 (13.6%)	Yes	44 (18.2%)
T-stage at presentation		No	198 (81.8%)
T1	169 (64.8%)	Chemotherapy	
T2	92 (35.2%)	Neoadjuvant chemo	46 (19%)
Pathologic grade		Early initiation of chemo	235 (97.1%)
G1	46 (19%)	CCRT	69 (28.5%)
G2-3	177 (73.1%)	Chemotherapy cycle≥3	198 (81.8%)
Gx	19 (7.9%)	RT	
T-stage at surgery		Type of RT	
T1	137 (56.6%)	ERBT	208 (86%)
T2	105 (43.4%)	ERBT+BT	34 (14.0%)
No. of removed nodes	21 ± 8.4	Technique	
No. of positive nodes	2 ± 3.82	3D-CRT	69 (28.5%)
Positive LN ratio (%)	15.5 ± 15	IMRT	173 (71.5%)
No. of positive LN		Median radiation intensity, (range)	
≥3	85 (35.1%)	Total dose (Gy)	50.4 (16-64)
<3	157 (64.9%)	No. of fractions	23-33
Pathologic tumor size		BT	
<4 cm	132 (50.6%)	Total dose^a^ (Gy)	24 (10-24)
≥4 cm	129 (49.4%)	No. of fractions	4 (2-4)
DTI			
Yes	228 (94.2%)		
No	14 (5.8%)		

^a^Brachytherapy dose.

Among the 242 patients, 235 initiated chemotherapy before RT, with a median interval of 10 (5–48) days between surgery and chemotherapy, 88% within 3 weeks, with 1–4 cycles (median: 1 cycle). The median interval between surgery and the initiation of RT was 38 (28–96) days.

Among the 235 patients with early initiated chemotherapy before radiotherapy, 48 patients continued chemotherapy every 3 weeks during radiotherapy; the other 187 patients continued the chemotherapy 2–4 weeks after radiotherapy. There were another 21 patients who had concurrent chemotherapy with weekly cisplatin, so totally 69 patients had CCRT.

Adjuvant chemotherapy mainly consisted of platinum-based chemotherapy (which included carboplatin plus paclitaxel in 66 patients, carboplatin plus docetaxel in 31, cisplatin plus paclitaxel in 93, cisplatin plus nab-paclitaxel in 24, and nedaplatin plus paclitaxel in 10), and 24 patients received other regimens.

### Efficacy

**Figure 1:**
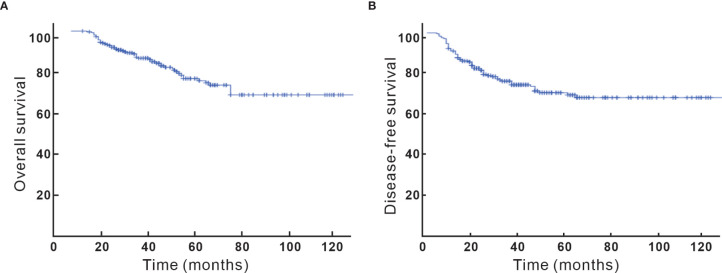
**(A)** Overall survival. **(B)** Disease-free survival.

After a median follow-up of 40 (5–132) months, 64 (26.4%) of the patients recurred and 51 (21.1%) died. Locoregional, para-aortic, and distant failure accounted for 13.2%, 7%, and 12%, respectively. The 5-year OS and DFS were 74% and 70%, respectively (**Figure 1**). Looking at factors predicting DFS, we observed that histology, tumor size, T stage at diagnosis, pT stage, parametrium invasion status, margin status, the burden of metastatic LN, early initiation of adjuvant chemotherapy or not, and neoadjuvant chemotherapy or not were associated with DFS at univariate analysis. *Via* multivariate analysis, only non-SCC, T2, ≥ 3 pLNs, and neoadjuvant chemotherapy were correlated with worse DFS, which is detailed in **Table 2**.

**Table 2:** Factors predicting disease-free survival in LN involved early-stage cervical cancer.

	Univariate analysis		Multivariate analysis	
	HR (95% CI)	P value	HR (95% CI)	P value
Squamous *vs* non-squamous	2.3 (1.31-4.17)	0.004	2.33 (1.25-4.33)	0.007
T1 *vs* T2 at presentation	2.75 (1.68-4.51)	0.000	2.10 (1.20-3.67)	0.009
<4cm *vs* ≥4cm at presentation	1.87 (1.09-3.19)	0.023		
Pathologic tumor diameter <4cm *vs* ≥4cm	1.39 (0.85-2.27)	0.191		
Pathologic T1 *vs* T2	2.52 (1.52-4.18)	0.000	1.91 (1.11-3.27)	0.019
L*vs*I: no *vs* yes	0.66 (0.40-1.08)	0.100		
Parametrium invasion: no *vs* yes	2.29 (1.34-3.92)	0.002		
Deep tumor invasion: no *vs* yes	2.18 (0.54-8.98)	0.274		
Margin status: negative *vs* positive	2.34 (1.34-4.07)	0.003		
The number of positive LN: <3 *vs* ≥3	1.99 (1.21-3.24)	0.006	2.04 (1.21-3.42)	0.007
Neoadjuvant chemotherapy: no *vs* yes	2.99 (1.79-4.98)	0.000	2.65 (1.54-4.54)	0.000
Early initiated chemotherapy before RT: no *vs* yes	2.11 (1.00-4.42)	0.049		
Concurrent chemotherapy: no *vs* yes	0.87 (0.49-1.55)	0.626		

When focused on OS, histology, T stage at presentation, pT stage, parametrium invasion status, margin status, the number of positive LN, and neoadjuvant chemotherapy or not were found to be correlated with OS at univariate analysis. *Via* multivariate analysis, non-SCC, T2, ≥ 3 LN metastasis, and neoadjuvant chemotherapy were correlated with worse OS, detailed in **Table 3**.

**Table 3:** Factors predicting overall survival in LN involved early-stage cervical cancer.

	Univariate analysis		Multivariate analysis	
	HR (95% CI)	P value	HR (95% CI)	P value
Squamous *vs* non-squamous	3.12 (1.71-5.71)	0.000	2.95 (1.66-5.24)	0.001
T1 *vs* T2 at presentation	2.22 (1.28-3.85)	0.004	1.89 (0.99-3.60)	0.051
<4 cm *vs* ≥4 cm at presentation	1.68 (0.94-3.02)	0.080		
Pathologic tumor diameter <4cm *vs* ≥4cm	1.26 (0.72-2.18)	0.416		
Pathologic T1 *vs* T2	2.53 (1.44-4.47)	0.001	1.99 (1.11-3.58)	0.022
L*vs*I: no *vs* yes	0.67 (0.38-1.16)	0.149		
Parametrium invasion: no *vs* yes	2.58 (1.42-4.66)	0.002		
Deep tumor invasion: no *vs* yes	1.99 (0.48-8.20)	0.340		
Margin status: negative *vs* positive	2.45 (1.32-4.53)	0.004		
The number of positive LN: <3 *vs* ≥3	2.14 (1.23-3.73)	0.007	2.14 (1.19-3.86)	0.011
Neoadjuvant chemotherapy: no *vs* yes	2.82 (1.60-4.98)	0.000	2.47 (1.35-4.53)	0.004
Early chemotherapy before RT: no *vs* yes	1.67 (0.78-3.54)	0.185		
Concurrent chemotherapy: no *vs* yes	1.07 (0.56-2.07)	0.834		

### T-Stage

**Figure 2:**
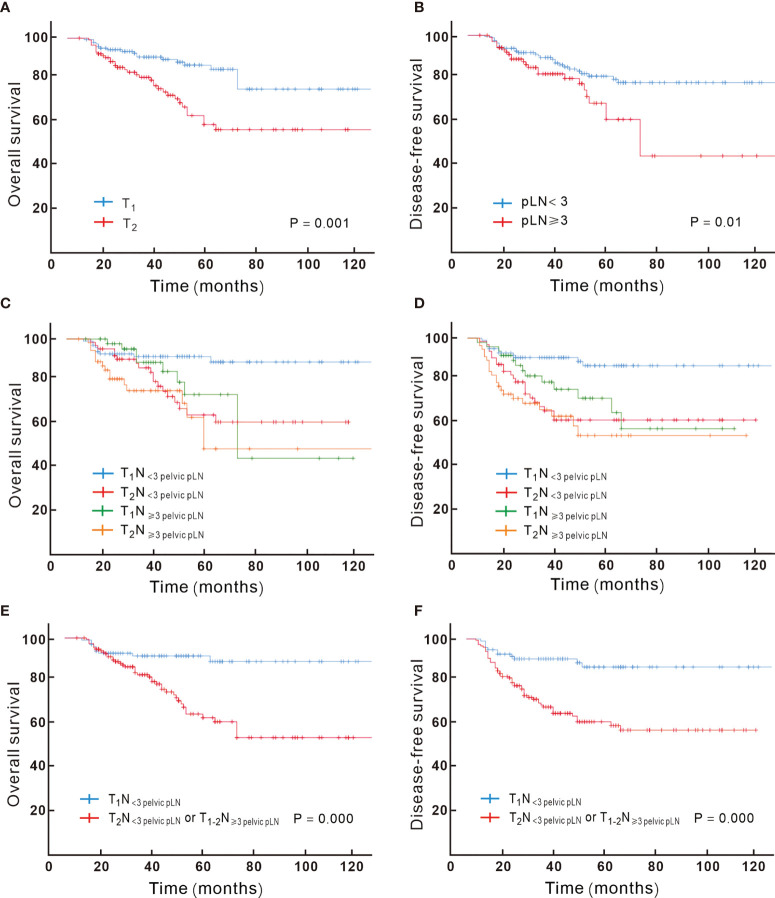
Kaplan–Meier analysis of overall survival for patients showed significant difference when stratified by T-stage **(A)** and the number of pathologic lymph node **(B)**; the overall survival and disease-free survival based on different combination of T and N substage are showed in **(C, D)**, with T_1_N_< 3 pelvic pLN_ showing significant better overall **(E)** and disease-free survival **(F)** compared to T_2_N_< 3 pelvic pLN_ or T_1-2_N _≥ 3 pelvic pLN_.

Via univariable analysis, DFS and OS varied significantly depending on the T-stage. Compared to T1, T2 patients had poorer prognosis. Out of the 105 T2 patients, 40 (38.1%) failed during the follow-up compared to 24 in 137 (17.5%) T1 patients (P = 0.000). The T2 group had higher risk of locoregional recurrence (8.0% *vs* 20%, P = 0.007). For T1 patients, 5-year OS was 86%, and for T2 patients, it was 58%, with an absolute survival difference of 28% (P = 0.001). On multivariable analysis, the T-stage remained an independent prognostic factor for DFS and OS (P = 0.000, **Figure 2A**).

### The Number of pLN

Patients with LVSI and non-SCC were more likely to have higher pLN burden (detailed in **Supplement Table 1**). The number of pLNs correlated with OS; ≥ 3 pLNs were associated with poor prognosis; 36.5% *vs* 21% recurred in ≥ 3 and < 3 pLN groups, respectively (P = 0.014), with 5-year OS of 59% *vs* 80% (P = 0.006, detailed in **Figure 2B**), and ≥ 3 pLNs was associated with more para-aortic failure (detailed in **Supplement Table 2**). For patients with ≥3 pLNs, no significant difference in the para-aortic recurrence risk was found between extended field irradiation (EFI) or not (P > 0.5); totally 3 patients recurred in the para-aortic region among 26 patients (11.5%) with EFI, and 8 recurred in 59 patients (13.6%) with pelvic irradiation.

### Combination of the Tumor and Node (TN) Category

**Figure 3:**
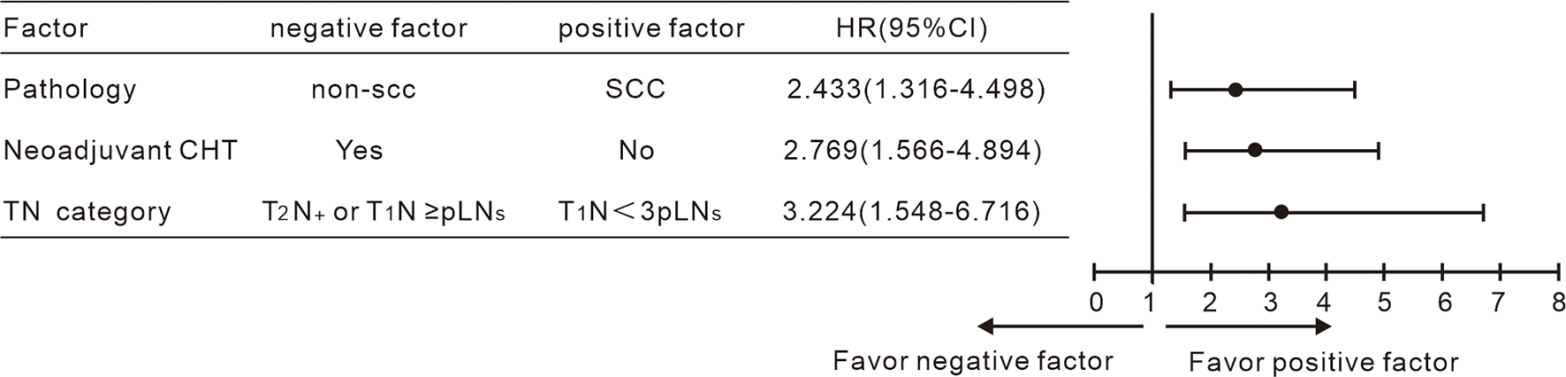
Multivariate Cox analysis including the TN category shown non-SCC, neoadjuvant chemotherapy and T_2_N_+/_T_1_N _< 3 pLN_ to be negative indicators for OS.

According to the results achieved by the multivariate Cox hazard model, we noted that the T-stage and the number of pLN strongly correlated with OS. We subclassified the N1 stage based on the number of pLNs; patients with <3 pLNs were defined as N_< 3 pLN_ and ≥ 3 pLNs as N_≥ 3 pLN_. Next, we regrouped the stage with different T and N combination and found that T1N_< 3 pLN_ had significantly different OS compared to the other groups, while T2N_< 3 pLN_, T1N_≥ 3 pLN_, T2 N_≥ 3 pLN_ had similar survival. When T2N_< 3 pLN_, T1N_≥ 3 pLN_, and T2N_≥ 3 pLN_ were grouped together, they had significantly inferior OS compared to T1N_< 3 pLN_, with 5-year OS of 92% vs 62%, respectively (P = 0.000, **Figures 2C–F**). This new classification also correlated with DFS, distant metastasis free survival (DMFS), and locoregional failure free survival (LRFS) (P < 0.05). A new multivariate Cox analysis including the new TN category, histology, T stage at presentation, pT stage, parametrium invasion status, margin status, the number of positive LNs, and neoadjuvant chemotherapy or not (all of which were with P < 0.05 *via* univariate analysis) was done. It was shown that non-SCC, neoadjuvant chemotherapy, and T2N_+_/T1N_≥ 3 pLN_ were negative prognostic factors for OS (**Figure 3**). Patients with non-SCC had significantly inferior outcome in T2N_+_/T1N_≥ 3 pLN_ (P = 0.002) but was not in the T1N_< 3 pLN_ group (P > 0.5).

### The Modality of Chemotherapy

Neoadjuvant chemotherapy was given to 46 patients, and 35 among the 46 patients (76.1%) were clinical T2 before neoadjuvant chemotherapy, with a mean tumor size of 6.7 cm. After neoadjuvant chemotherapy, the mean tumor size reduced to 3.6 cm, with 24 pathologic (52.2%) T2. A total of 19 patients (41.3%) had clinical metastatic LN at presentation. Patients with neoadjuvant chemotherapy were more likely to have larger tumor and advanced disease at presentation (P = 0.000). About 71.4% of the patients were T2 in the neoadjuvant group at presentation compared to 26.9% T2 in the direct surgery group (P =0.000). Patients with neoadjuvant chemotherapy had poorer OS compared to the direct surgery group (P = 0.000).

During radiotherapy, 69 patients had concurrent chemotherapy. We further analyzed if the addition of concurrent chemotherapy could improve survival outcome. No significant difference of OS between the addition of concurrent chemotherapy or not was found (P > 0.5).

### Toxicity

No treatment-related death was observed. Four (1.7%) patients discontinued radiotherapy. One of the patients received one cycle of chemotherapy before radiotherapy and had weekly cisplatin during radiotherapy; she discontinued RT at 16 Gy due to grade 2 gastrointestinal toxic effects. One patient received two cycles of chemotherapy before radiotherapy, and due to grade 3 gastrointestinal toxic effects, she stopped RT at 27 Gy; another patient discontinued RT at 30 Gy due to incomplete ileus, and she did not receive any chemotherapy; and the rest received one cycle of neoadjuvant chemotherapy, one cycle chemotherapy before radiotherapy, and ceased RT at 37.8 Gy also due to grade 3 gastrointestinal toxic effects.

The incidence of grade 3 or 4 hematological toxic effects was 14.1% during radiotherapy; the addition of concurrent chemotherapy significantly increased the hematological toxicity, with 34.3% *vs* 7% of grade 3 or 4 leukopenia in the CCRT and no CCRT groups, respectively (P = 0.000).

During the long-term follow-up, two patients had grade 2 lower limb edema (LLE), and two patients had grade 3 LLE (CTCAE v5.0, Adverse Event Edema: limb).

Seven patients developed severe urinary toxicity in the long-term follow-up. Two patients had ureteral stricture that needed repeated ureteral stenting, and another five patients had bladder dysfunction, manifested as storage dysfunction, voiding dysfunction, or stress urinary incontinence, and could not be controlled by medication, which needed a pacemaker or catheterization.

## Discussion

Stage C1 reflects a heterogeneous group of tumors with a wide range of survival, and there may be some problem to give the same treatment to patients with different recurrence risk. Our cohort showed that the T-stage and the number of pLNs (≥ 3 or < 3) were all important prognostic factors in early-stage cervical cancer with LN involvement; the combination of the T-stage and N substage (based on ≥ 3 or < 3 pelvic pLNs) had better prognostic discrimination. Patients with T_1_N _< 3pLN_ had a predicted 5-year OS of 92% after SCRT following radical hysterectomy compared with 62% in the other patient group.

LN involvement was associated with an inferior outcome across all stages; the presence of lymphatic spread correlated to 10–30% reduction in 5-year survival outcomes (10). In the analysis of the SEER database, survival of patients with pelvic pLN significantly differed based on the T-stage, with a 35.5% difference in absolute OS (5-year OS rate of 74.8% for T1, 58.7% for T2, and 39.3% for T3, P = 0.001) (6). In the subgroup analysis from NCDB, when stratified by nodal status, there was generally a decrease in survival with increasing T-stage (11); the 5-year OS was 80.3% in T1 with pelvic pLN compared with 57.2% in T2 (12). In the current study, we found that the T-stage was a main prognostic factor, with a predicted difference of 28% at 5-year OS between T1 and T2 (86% and 58%, respectively).

LN burden was another important prognostic factor in cervical cancer, with the number of pLN ≥ 3 to be a negative prognostic factor for OS compared to < 3. Nodal staging system based on the number of pLNs is widely used in other cancers such as breast, stomach, and rectum. To date, data on burden of nodal disease in cervical cancer are limited. In studies of patients with early-stage cervical cancer treated with radical surgery followed by radiotherapy, some authors showed that the survival difference between patients with 1 and ≥ 2 pelvic pLN(s) was statistically significant (8). On the other hand, Tsai et al. (13) reported that patients with only one pLN had achieved similar outcomes compared to pN0, and patients with ≥ 2 pelvic pLNs had lower survival rates (87% *vs*. 61%, p < 0.001) than those with pN0. In the study from Bogani et al. (14), median disease-free survival was 100, 42, and 12 months for patients with one, two, and three or more positive node(s), respectively. Kwon et al. evaluated a group of 249 cervical cancer patients treated in 13 Korean institutions (15). They observed that the presence of pLNs (≥ 4) was an independent predictor for DFS. Besides the number of pLN, the LN ratio was also suggested to be associated with DFS and OS in some studies (16–18). Possible explanation of the observed difference in the number of pelvic pLNs will be that the risk of recurrence, especially distant failure, might be attenuated by concurrent or consolidation chemotherapy. In the current study, the risk of distant metastasis was comparable between ≥ 3 and < 3 pLNs, but higher risk of para-aortic recurrence was observed in patients with ≥ 3. For patients with ≥ 3 pLNs, 11.3% and 13.5% of patients recurred in the para-aortic area with or without EFI; the risk could not be attenuated by EFI. Prophylactic EFI was believed to be evaluable for patients with high risk of para-aortic LN recurrence in patients without chemotherapy, but in the era of CCRT, whether patients can benefit from prophylactic EFI is still controversial (19). In the current study, all of the patients had adjuvant chemotherapy, which may also nullify the effect of EFI in preventing para-aortic LN recurrence. Given these findings, inclusion of all women with nodal disease in one stage category may not provide enough prognostic precision to be clinically meaningful. Staging systems that combine local tumor characteristics and nodal status to assign a stage are already widely used for other solid tumors. In an exploratory analysis, we reclassified N1 into N_< 3 pLN_ and N_≥ 3 pLN_. It was found that the combination of T and N substage resulted in improved prognostic discrimination, with T1N_< 3 pLN_ having significantly better survival compared to T_2_N_< 3 pLN_/T_1-2_N_≥ 3 pLN_, with recurrence rates of 11.6% and 35.8% in each group, and the predicted 5-year OS was 92% and 62%, respectively. Non-SCC, mainly adenocarcinoma, is considered to be a negative prognostic factor in uterine cervical cancer (20). And patients with non-SCC in the T2N_+_ and T1N_≥ 3pLN_ group had poorer outcome compared to SCC, but not in the T1N_< 3 pLN_ group, which may reflect the possible interaction between pathology and the tumor stage. Tumor size is also considered an independent prognostic factor (21), and we also analyzed the interaction between tumor size and the TN stage; but no difference in the outcome was found between different size (data not shown here). This may be due to the trimodality therapy (surgery, RT, and chemotherapy) here, which may result in better local and even distant control.

Treatment options are often determined tailored to the stage and prognosis; it seems unreasonable to give the same treatment to patients with significantly different prognosis. Radical CCRT is recommended for patients with radiologic or intraoperatively detected positive LN, but the management of patients with LN involvement remains controversial in clinical practice, with surgery remaining the main treatment in reports. Several studies together showed that 5-year OS was equivalent between radical hysterectomy and radical CCRT, with different treatment-related morbidity in each group. **Supplement Table 3** showed studies focused on early-stage cervical cancer with high risk factors; most of them were with positive LN (7, 12, 14, 17, 22–29). Radiotherapy was given after radical surgery in most of these studies, with variable combination of chemotherapy, such as CCRT, concurrent and consolidation chemotherapy, and SCRT (22–24, 30, 31). And SCRT following surgery was reported to be superior than CCRT in disease control for patients with high-risk factors (23, 30). All of the patients in our cohort had SCRT. It was satisfactory to have a 5-year OS of 92% for patients with T_1_N_< 3 pLN_ in our cohort, which was comparable to that in the STARS study (23) and studies from Bogani et al. (14) and Tim et al. (25). From this point, radical surgery followed by SCRT was a reasonable treatment option for this group of patients.

But for patients with T_2_ N_< 3 pLN_/T_1-2_ N_≥ 3 pLN_, survival outcomes remained unsatisfactory. The predicted 5-year OS was 62% in the current cohort for these patients. In studies that used surgery as the main treatment (6, 8, 11, 14), the 5-year OS ranged from 55.3% to 57.2%, which was even poorer than the current cohort. In studies that used radical radiotherapy as the main treatment, the outcome was not better. In the RetroEMBRACE study (32), patients were treated with radical radiotherapy; among the patients with positive LN, 27% of systemic failure, 10.2% of para-aortic, 8.2% of regional failure, and 11.5% of local failure were reported. A more detailed outcome based on the T-stage in the positive LN group was not mentioned in this study. In the study from Ryu et al., the 5-year OS for III C1r with parametrium invasion was 65% after radical chemoradiotherapy (33). And the 3-year DFS for patients with ≥ 2 LN involvement was 51.1% after radical radiotherapy in Liu’s study (34). In conclusion, the outcome was poor after either surgery or radical radiotherapy. In our cohort, for patients with T2N_+_ or T1N_≥ 3 pLNs_, 35.8% had treatment failure; distant and locoregional failures were the major problems. Since the patients all had received intensified treatment, this raises the question of whether these patients should be considered for other innovative therapies, thus potentially increasing their survival outcomes. Studies evaluating the role of systemic treatments including standard chemotherapy plus immunotherapeutic agents are needed. Additionally, further prospective randomized studies are warranted in order to identify effective treatment modalities in this group of patients.

Besides efficacy, another major concern of treatment was treatment-related toxicity. Impaired social functioning, less sexual enjoyment, and higher symptom experience scores were reported more after primary (C)RT compared with patients with adjuvant (C)RT; and LLE was reported more in the surgery group (35). Though trimodality (surgery followed by chem-radiotherapy) was given to the patients in our cohort, toxicity was acceptable. About 1.5% discontinued radiotherapy due to G2-3 gastrointestinal toxicity. The patient-reported G2 and G3 LLE rate was low, with an incidence total of 1.7%. Urinary tract morbidity is the most common long-term complication of radical surgery, and the addition of radiotherapy may aggravate this situation. Severe urinary toxicity was observed in 2.9% of the patients, manifested as ureteral stricture or bladder dysfunction.

The inherent biases of the single-center retrospective study design represented the main weakness of the paper, while the large sample size, the homogeneity of adjuvant treatment, and the long-term follow-up represented the main strengths of the present research. It was difficult to retrospectively evaluate posttreatment morbidity, especially long-term adverse events after adjuvant therapy. And we also could not distinguish macrometastasis, micrometastasis, and pLN with isolated tumor cells in this retrospective study. The study supports conclusions drawn from previous retrospective studies that a well-powered randomized controlled trial is needed to compare both oncological and QoL outcomes for the preferred treatment of lymph node-positive early-stage cervical cancer, and to verify the TN subcategory in predicting outcomes.

## Conclusion

Nodal involvement alone is inadequate as the sole pathologic factor to predict survival in early-stage cervical cancer, and the combination of the T stage and the number of pelvic pLNs provides better prognostic discrimination.

## Data Availability Statement

The original contributions presented in the study are included in the article/**Supplementary Material**. Further inquiries can be directed to the corresponding author.

## Ethics Statement

The studies involving human participants were reviewed and approved by the Institutional Review Board of Renji Hospital. The patients/participants provided their written informed consent to participate in this study.

## Author Contributions

HC took part in the conceptualization, project administration, and writing, reviewing, and editing the manuscript. YB took part in the conceptualization, data curation, and writing and preparation of the original draft. LR took part in the formal analysis, investigation, and data curation. BH took part in the formal analysis, investigation, and data curation. JW took part in the telephone interview. XM was in charge of the software and formal analysis. All authors contributed to the article and approved the submitted version.

## Funding

The present study was supported by the National Natural Science Foundation of China (grant no. 81903129 to HC) and the Beijing Xisike Clinical Oncology Research Foundation (Y-XD202001/zb-0011 to YB).

## Conflict of Interest

The authors declare that the research was conducted in the absence of any commercial or financial relationships that could be construed as a potential conflict of interest.

## Publisher’s Note

All claims expressed in this article are solely those of the authors and do not necessarily represent those of their affiliated organizations, or those of the publisher, the editors and the reviewers. Any product that may be evaluated in this article, or claim that may be made by its manufacturer, is not guaranteed or endorsed by the publisher.
