# Supplementary material for: The Combination of T Stage and the Number of Pathologic Lymph Nodes Provides Better Prognostic Discrimination in Early-Stage Cervical Cancer With Lymph Node Involvement
Source: Front Oncol. 2021 Nov 5;11:764065. doi: 10.3389/fonc.2021.764065 (PMC8602848; doi:10.3389/fonc.2021.764065)
Supplement: Supplementary file 1 [file DataSheet_1.zip › Supplement Materials-revision.docx]

Supplement Tab.1 The relationship between the number of pathologic LN and other pathology characteristics

|  | <3 LN(s) | ≥3 LNs | P value |
| --- | --- | --- | --- |
| Pathology | | | |
| SCC | 144 | 82 | 0.025 |
| Non-SCC | 15 | 20 |  |
| Pathologic tumor size | | | |
| <4 cm | 82 | 40 | 0.501 |
| ≥4 cm | 75 | 45 |  |
| DTI | | | |
| Yes | 147 | 81 | 0.775 |
| No | 10 | 4 |  |
| LVSI | | | |
| Yes | 100 | 66 | 0.030 |
| No | 57 | 19 |  |
| Margin status | | | |
| ≥5mm | 134 | 72 | 1.0 |
| <5mm | 23 | 13 |  |
| Parametrial invasion | | | |
| Yes | 24 | 20 | 0.119 |
| No | 133 | 65 |  |
| T stage | | | |
| T1 | 95 | 42 | 0.105 |
| T2 | 62 | 43 |  |

Supplement Tab.2 The difference of failure pattern between patients with < 3 and ≥ 3 pLNs

|  | <3 LNs | ≥3 LNs | P value |
| --- | --- | --- | --- |
| Locoregional | 17 (10.8%) | 15 (17.6%) | 0.164 |
| Para-aortic | 6 (3.8%) | 12 (14.1%) | 0.015 |
| Distant | 17 (10.8%) | 11 (14.2%) | 0.535 |
| Total | 33 (21.0%) | 31 (36.5%) | 0.014 |

Supplement Tab.3: Studies focused on early-stage cervical cancer with LN involvement

|  | case | stage | Risk factor | treatment | Follow up | Recurrence rate | OS(5yr) | Toxicity (G3-4) |
| --- | --- | --- | --- | --- | --- | --- | --- | --- |
| Richard et al 2007 ^(30)^ | 218 | IB | LN+ | ①Surgery + CCRT  ②CCRT | 77 or  69 m | NR | 76%  69% | NR |
| Ziebarh et al 2012 ^(31)^ | 41 | IB | LN+ | ①Surgery (+ RT 73%)  ②RT | 42.3 m | 30.8%  60% | 80%  65% | 26.9%  46.7% |
| Derks et al 2017 ^(32)^ | 121 | ⅠB-ⅡA (90.1% IB) | LN+ | ①Surgery (+RT±CT )  ②CCRT | 32 m | (5y DFS)  81%  67% | 84%  77% | 30%  59% |
| Cibula et al 2021 ^(4)^ | 515 | ⅠA-ⅡB (73.9% I) | LN+ | ①Surgery (+CCRT 74%, + RT 13%, + CT 5%)  ②CCRT | 58 m | 25.8%  26.6% | 72%  69% | NR |
| Peters et al 2000 ^(33)^ | 243 | ⅠA2-ⅡA (94% ⅠB) | High risk (85.2% LN+) | Surgery +  ①RT  ②CCRT+CT | 42 m | (4 yr DFS)  63%  80% | (4 yr)  71%  81% | 3.6%  17.2% (G4) |
| Trifiletti et al 2015 ^(12)^ | 3053 | T1-T4 (T1 60.2% T2 31.3%) | High risk (77% LN+) | Surgery +  ①RT+CT  ②RT alone (19%) | NR | NR | 73.3%  67.6% | NR |
| Zhao et al 2017 ^(26)^ | 146 | ⅠA2-ⅡB (58.1% Ⅰ) | High risk (71.5% LN+) | Surgery+  ①CT+RT  ②CCRT | 30 m | 21.3% | (3 yr)  86.6%  78.3% | Hematologic:  12.3%  25.4% |
| Kim et al 2017 ^(27)^ | 98 | ⅠA2-ⅡA (97% ⅠB) | High risk (80.6% LN+) | Surgery + CT + CCRT +CT | 119 m | 13.3% | 88.7% | Hematologic: 37.8%,  Gastrointestinal: 14.3% |
| Bogani et al 2019 ^(14)^ | 177 | ⅠA-ⅡB  (44.6% ≤ⅡA1) | LN+ | Surgery+  ①RT (47.5%)  ②CCRT (39.5%)  ③CT (13%) | 58 m | 37.3% | 91%^a^; 75%^b^ | NR |
| Kim et al 2020 ^(7)^ | 483 | ⅠA-ⅡA  (82% IB) | High risk (74.5% LN+) | Surgery + CCRT | 57 m | 25.3% | 82.5% | NR |
| Aslan et al 2020 ^(17)^ | 185 | IB1–IIA2 | LN+ | Surgery + CCRT | 45.5 m |  | 70.4% | NR |
| Huang et al 2021 ^(23)^ | 273 (whole group 1048) | ⅠB1-ⅡA2 | High risk (86.1% LN+) | Surgery +  ①SCRT  ②CCRT  ③CT | 56 m | (3 yr DFS)  92%  77%  77% | NR | 25.3%  28.5%  12.3% |

Supplement Fig.1 Disease free survival varied based on T stage (A) and the number of pathologic lymph node (B).

Supplement Fig.2 Patients with T_1_N _<3 pelvic pLN_ showed significant better distant metastasis-free survival (A) and locoregional failure-free survival (B) compared to T_2_N _<3 pelvic pLN_ / T_1-2_N_≥3 pelvic pLN_.
